# *Helicobacter pylori* promotes apoptosis, activates cyclooxygenase (COX)-2 and inhibits heat shock protein HSP70 in gastric cancer epithelial cells

**DOI:** 10.1007/s00011-012-0487-x

**Published:** 2012-05-19

**Authors:** Aneta Targosz, Tomasz Brzozowski, Piotr Pierzchalski, Urszula Szczyrk, Agata Ptak-Belowska, Stanislaw Jan Konturek, Wieslaw Pawlik

**Affiliations:** 1 Department of Physiology, Jagiellonian University Medical College, 16 Grzegorzecka Str., 31-531 Cracow, Poland; 2 Department of Medical Physiology, Faculty of Health Sciences, Jagiellonian University Medical College, Cracow, Poland

**Keywords:** *Helicobacter pylori*, Apoptosis, CagA, Heat shock protein 70, Cyclooxygenase-2

## Abstract

**Objective:**

Apoptosis plays an important role in the regulation of gastric epithelial cell number and gastrointestinal disorders induced by *Helicobacter pylori* (*Hp*). Heat shock proteins (HSPs) are involved in cell integrity, cell growth and in gastric mucosa colonized by *Hp*. COX-2 was implicated in *Hp*-induced carcinogenesis but the effects of this germ and CagA cytotoxin on HSP70, COX-2, Bax and Bcl-2 in gastric cancer epithelial cells have been little studied.

**Material and methods:**

We determined the expression for HSP70, Bax and Bcl-2 in human gastric epithelial MKN7 cells incubated with live strain *Hp* (*cagA* + *vacA*+) with or without co-incubation with exogenous CagA and NS-398, the selective COX-2 inhibitor. After 3–48 h of incubation, the expression of HSP70, COX-2, Bax and Bcl-2 mRNA and proteins were determined by RT-PCR and immunoprecipitation.

**Results:**

*Hp* inhibited expression for HSP70 and this was significantly potentiated by exogenous CagA. Co-incubation of epithelial cells with *Hp*, without or with CagA increased Bax expression and simultaneously decreased expression for Bcl-2. The increase in COX-2 mRNA and Bax expression were significantly inhibited by NS-398. We conclude that Hp promotes apoptosis in adenocarcinoma gastric epithelial cells *in vitro* and this is associated with activation of COX-2 and inhibition of HSP70.

## Introduction

The gastric mucosa integrity depends on a balance between the renewal and the death of damaged or aged cells. Apoptosis, first proposed by Kerr et al. [1] in 1972, plays a complementary but opposite role to that of cell proliferation in regulation of normal growth and development of animal and human tissue. Gastric mucosa shows a high rate of renewal. The surface mucosal cells are constantly exfoliating into the gastric lumen, with a 3–5 day renewal rate under normal physiological conditions. Apoptosis has been reported to take place in all regions of stomach, occurring predominantly in the upper part of the gastric glands and involving 2–3 % of all epithelial cells [2, 3]; however, this process in gastric cancer cells has been little investigated.

The regulation of apoptosis is a complex process that includes the activation of various apoptosis-related proteins such as the Bcl-2 family, p53, Fas and its ligand (FasL). The Bcl-2 family is one of the most thoroughly studied groups of proteins involved in the regulation of apoptosis under normal conditions. Some members of the Bcl-2 family, such as Bax, Bad or Bid promote apoptosis, whereas the other members, such as Bcl-2 and Bcl-x_l_ were shown to inhibit this process [4–6]. Many bacterial pathogens have been identified as factors influencing the process of apoptosis in vivo and in vitro. Among the bacterial factors, the most important is *Helicobacter pylori* (*Hp*), which is a spiral, Gram-negative microaerophilic bacterial pathogen that has been categorized by WHO as a class I carcinogen, infecting the stomach of about 50 % of the world’s population [7, 8]. At present, the mechanisms by which *Hp* affects apoptosis in gastric epithelial cells still remains unclear because of the lack of availability of normal human epithelial cell line suitable to study these aspects in vitro conditions.

The major disease-associated genetic difference in *Hp* induced the upper gastrointestinal disorders is the presence (*cag+* and *vacA*+) or absence (*cagA−* and *vacA−*) of the pathogenicity island (*cag*-PAI), which is a locus of about 40-kilobase containing up to 27–31 genes [9, 10]. One strain-specific *Hp* locus that has been associated with an increased risk of carcinogenesis is the *vacA* gene, which encodes a secreted bacterial cytotoxin identified as VacA. When added to mammalian cells in vitro, cytotoxin VacA induces multiple structural and functional alterations in the cell, the most prominent of which is the formation of large intracellular vacuoles [11–13]. Cytotoxin-associated gene A (*cagA*) is the only known *Hp*-protein translocated into host cells followed by tyrosine phosphorylation through host protein kinases. *Hp* strains possessing the *cagA* gene has been linked to an increased risk of the development of peptic ulceration and gastric cancer in infected individuals. However, this protein is not critical to the pathogenesis of the disease, due to the fact that many strains, which possess CagA do not cause disease and some strains that are associated with disease do not express *cagA* [14, 15]. The *cag-*PAI encodes a type IV secretion system (TFSS) that forms a syringe-like structure, which is used to translocate the bacterial proteins into different types of eukaryotic cells. Once introduced into the host cells, bacterial toxins promote the synthesis and secretion of chemokines, such as interleukin-8 (IL-8) [16, 17]. Moreover, TFSS activates intracellular signaling including NFκB and specific kinases such as mitogen-activated kinase (MAPK), that subsequently promote the transcription of genes responsible for inflammation, proliferation or apoptosis [18].

Heat shock proteins (HSPs) represent a nearly universal phylogenetically and highly conserved family of proteins that form the machinery able to prevent damage to the cell structure from a variety of environmental stresses and unfavorable conditions. HSPs act as molecular chaperons folding newly synthesized cell proteins and assisting in the refolding of damaged proteins. In particular, HSP70 is located in all cellular compartments including the mitochondria, endoplasmic reticulum, cytosol and nucleus [19–21]. HSP70 can be produced in response to environmental changes such as heat, ischemia, heavy metals, ethanol, toxin, oxidants, various viral and bacterial infections and it is believed that their primary function is to protect the body’s cells against stress [22].

Cyclooxygenase (COX), the rate-limiting enzyme in the conversion of arachidonic acid to prostaglandin H_2_, is the main target of non-steroidal anti-inflammatory drugs (NSAIDs). Two isoforms of this enzyme have been identified: COX-1 and COX-2. The isoform COX-1 is constitutively expressed in most tissues and is involved in the production of prostaglandins (PG) to maintain normal physiological functions. COX-2 is involved in inflammation and triggered by mitogens, cytokines, hormones and growth factors. Several recent studies suggest that COX-2 might be an important factor in carcinogenesis, and COX-2 inhibitors were shown to possess anticancer effects [23]. Since *Hp* CagA and VacA positive and negative strains were reported to inhibit [24], and in some studies, to promote, the apoptosis in gastric mucosal cells in vivo and in vitro [25–29], we studied the effect of incubation with *Hp* of human MKN7 adenocarcinoma cell line with relation to expression HSP70, COX-2 and apoptosis. The purpose of our present study was several folds: (1) to determine the effect of live *Hp* strain expressing *cagA* and *vacA* on the expression of HSP70 in gastric epithelial MKN7 cells; (2) to examine the apoptosis rate in these cells by assessing the expression of mRNA for Bax and Bcl-2; (3) to compare the effect of cell incubation with *Hp* strain expressing cagA and vacA applied alone or in combination with exogenous CagA protein on the expression of HSP70, Bax and Bcl-2 in MKN7 cells; (4) to compare the effect of *Hp* strain expressing *cagA* and *vacA* and strains negative for *cagA* and *vacA* coincubated with or without the NS-398 on mRNA expression for COX-2 and apoptotic proteins Bax and Bcl-2 in MKN7 cells.

## Materials and methods

All experimental procedures performed in this study were run in accordance to the Helsinki Declaration and approved by the Jagiellonian University Institutional Animal Care and Use Committee.

### Bacterial strains and their characterization

Strains of *Hp* used in this study were isolated from gastric biopsy specimens of the patients with gastric ulcer who underwent upper endoscopy. The bacteria were grown on Columbia Agar supplemented with 5 % fresh horse blood (BioMerieux, Marcy l’Etoile, France). The plates were incubated under microaerophilic conditions at 37 °C for 3–5 days.

Genomic DNA was isolated from *Hp* strains obtained from patients using DNAzol Reagent (Life Technologies, NY, USA) according to the manufacturer’s protocol. For each single PCR reaction, 20 μg of DNA was used. Specific primers for the detection of *cagA* and *vacA* were synthesized by Sigma-Aldrich (St. Louis, USA). *CagA* and *vacA* positive and negative strains of *Hp* were used in experiments described in this study. Stock cultures were maintained at –70 °C in Brucella Broth supplemented with 10 % fetal bovine serum and 10 % glycerol. Prior to the incubation with MKN7 cells, bacterial strains of *Hp* were suspended in sterile PBS.

### Cell line and culture conditions

MKN7 human gastric carcinoma cells were grown in RPMI 1640 medium (Sigma-Aldrich, USA) supplemented with 10 % fetal bovine serum at 37 °C with 5 % CO_2_ and humidified atmosphere in absence or in the presence of *Hp* alone or in combination with the recombinant CagA (OraVax Inc., Cambridge, USA) or NS-398, a selective COX-2 inhibitor. Before the experiments cells were seeded on 100 mm culture dish in RPMI 1640 with addition of 2 % fetal bovine serum without antibiotics. MKN7 cells were infected with 1 × 10^9^ of live *Hp* per dish (calculated to approximately 300 *Hp* bacteria per cell) and co-incubated with 10 μg of CagA per 1 ml of RPMI medium or with 50 mM of NS-398.

### Determination of Bax and COX-2 expression by RT-PCR

After 3, 6, 24 and 48 h of incubation the cells were harvested and the total cellular RNA was isolated using Trizol Reagent (Invitrogen, Carlsbad, USA) according to the manufacturer’s protocol. Following precipitation, RNA was resuspended in RNase-free water and its concentration was estimated by the absorbance at 260 nm wavelength. The RNA integrity in each sample was confirmed by 1 % agarose-formaldehyde gel electrophoresis and ethidium bromide staining. Aliquoted RNA samples were stored at –80 °C until analysis. The synthesis of the first strand cDNA was performed with Reverse Transcription System (Promega, Madison, USA) using 2 μg of RNA. For the PCR, 2 μl of cDNA and oligo primers were used. All PCR reactions were carried out using a Promega PCR reagents. The DNA was amplified in the thermal cycler (Biometra T3, Berlin, Germany) with 20 cycles for β-actin (denaturation at 95 °C for 1 min, annealing 54 °C for 1 min and extension for 72 °C for 2 min), 29 cycles for Bax (denaturation at 94 °C for 45 s, annealing 55 °C for 45 s and extension for 74 °C for 2 min), 35 cycles for COX-2 (denaturation at 94 °C for 1.5 min, annealing 58 °C for 1.5 min and extension for 72 °C for 1.5 min). Specific primers were synthesized by Sigma-Aldrich (St. Louis, USA). The following human primers were used: β-actin s-5′AGC GGG AAA TCG TCG GTG 3′, a-5′GGG TAC ATG GTG GTG CCG 3′, Bax s-5′ TGG CAG CTG ACA TGT TTT CTG 3, a-5′ CGT CCC AAC CAC CCT GGT CT 3′, Bcl-2 s-5′ GAC AGC CAG GAG AAA TGA AA 3′, a-5′ GAC TTC TTC CGC CGC TAC 3′, COX-2s-5′ AGA TCA TCT CTG CCT GAG TA 3′, a-5′ CCT TCT TAA CCT CTC CTA TTA TA 3′.

PCR products were detected by electrophoresis on 2 % agarose gel containing ethidium bromide. Location of the predicted PCR products was confirmed using O’Gene Ruler 50 bp DNA Ladder (Fermentas Life Sciences, San Francisco, USA) as a standard-size marker.

### Immunoprecipitation

Protein extracts from MKN7 cells were prepared as described by Cha et al. [30]. Samples containing 10 μg of proteins were incubated for 4 h at 4 °C on a shaking platform with 5 μl of primary goat polyclonal anti HSP70, rabbit monoclonal anti Bax, and mouse polyclonal anti Bcl-2 antibodies. Five microliters of A-agarose were added to each sample and samples were incubated overnight at 4 °C. Complexes were washed three times with RPB-buffer (150 mM NaCl, 1 % NP-40, 0.5 % deoxycholate, 0.1 % SDS, 50 mM Tris pH 8). Then 10 μl of western blot sample buffer (0.1 M Tris–HCl pH 6.8, 4 % SDS, 20 % glycerol, 2 % mercaptoethanol, 0.2 % bromophenol blue) was added to each pellet, boiled for 5 min at 95 °C and loaded on the 12 % SDS-polyacrylamide gel. After electrophoresis and transfer of the samples, the PVDF membrane (BIORAD, Hercules, USA) was blocked with 5 % non-fat dried milk in PBS for 2 h at room temperature. Blocking procedure was followed with 1 h exposure to primary antibody diluted 1:1000 and secondary antibody anti goat, anti rabbit, and anti mouse diluted 1:1000 in blocking buffer. All antibodies were purchased from Santa Cruz Biotechnology (Santa Cruz, USA). After each antibody probing membrane was washed three times for 15 min in TBST buffer (0.1 M Tris Ph 8.0; 1.5 M NaCl; 0.5 % TritonX-100). Detection of membrane bound proteins was performed using West Pico Chemimiluminescent (Pierce, Rockford, USA).

### Statistical analysis

Results are expressed as means ± SEM. Statistical analysis was done using analysis on variance and two ways ANOVA test when appropriate. Differences with *p* < 0.05 were considered statistically significant.

## Results

### The effect of live Hp (cagA and vacA positive) strain with or without coincubation with exogenous CagA on the HSP70, Bax and Bcl-2 expression in MKN7 gastric epithelial cells

**Fig. 1:**
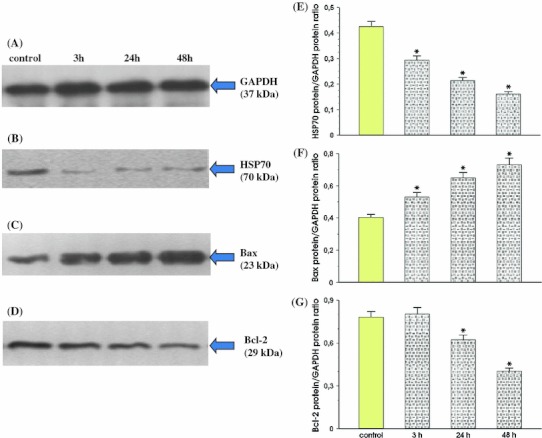
Immunoprecipitation analysis of HSP70, Bax, Bcl-2 in total cellular proteins isolated from MKN7 cells exposed to *Hp* expressing *cagA* and *vacA* (*left panel*) and densitometric analysis of the signal for expression of HSP70, Bax and BCl-2 protein. Total cellular proteins in the concentration of 10 μg of was loaded per each lane. Proteins extracts from control cells (*lane 1*) and cells coincubated with *Hp* over the time period of 3 h (*lane 2*), 24 h (*lane 3*) and 48 h (*lane 4*) were separated in 12 % SDS-polyacrylamide gel. Blots were probed with anti human HSP70, Bax, Bcl-2 antibody, respectively. Mean ± SEM of four determinations. *Asterisks* (*p* < 0.05) indicate a significant increase or decrease above the control values

**Fig. 2:**
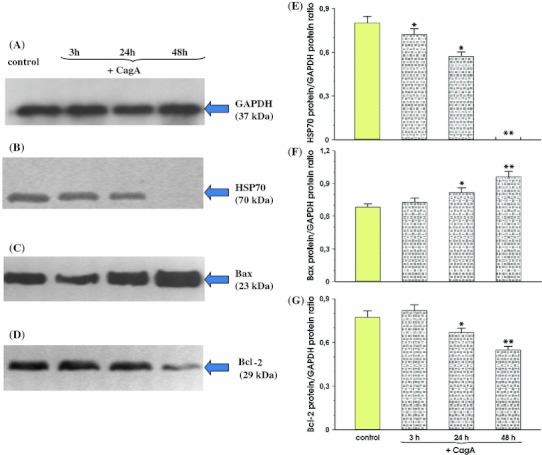
Immunoprecipitation analysis of HSP70, Bax, Bcl-2 in total cellular proteins isolated from MKN7 cells coincubated with *Hp* expressing *cagA* and *vacA* with addition of exogenous CagA. Amount of 10 μg of total cellular proteins was loaded per each lane. Proteins extracts from control cells (*lane 1*) and cells coincubated with *Hp* with addition of exogenous CagA over 3 h (*lane 2*), 24 h (*lane 3*) and 48 h (*lane 4*) were separated in 12 % SDS-polyacrylamide gel. Blots were probed with anti human HSP70, Bax, Bcl-2 antibody, respectively. Mean ± SEM of four determinations. *Asterisks* indicate a significant (*p* < 0.05) increase or decrease above the control values. *Double asterisks* indicate a significant (*p* < 0.05) increase or decrease above or below the values recorded at 3 and 24 h, respectively

In this series of experiments, the MKN7 cells were exposed to live bacteria (1 × 10^9^ per culture dish) over different periods of time starting from 3 h up to 48 h. As expected the basal expression of HSP70 and mRNA for COX-2 in MKN7 cells was relatively high due to the intensive growth rate of that kind of cell type. As shown in Fig. 1 panel b, the *cagA* and *vacA* positive *Hp* strain inhibited in a time-dependent manner the expression of HSP70 protein in MKN7 cells; the inhibitory effect on HSP70 was observed already at 3 h and the inhibitory effect of this strain of *Hp* on HSP70 expression was subsequently noted at 24 h and 48 h after exposure of cell culture to this bacteria. In the presence of *Hp* in the culture medium, the expression of HSP70 in MKN7cells was significantly downregulated at protein levels. Densitometry analysis confirmed that the expression of HSP70 protein in MKN7 cells was significantly decreased in cells coincubated with bacteria as compared to control cultures treated with saline instead of bacteria (Fig. 1, panel e). The expression for Bax increased 3 h after *Hp* infection, reaching its maximal level at 48 h of incubation. The expression of Bax was significantly increased when MNK7 cells were incubated with or without coincubation with recombinant CagA (Fig. 1c). Ratio of Bax protein over GAPDH protein confirmed that the expression of Bax protein was significantly increased when MNK7 cells were incubated with or without coincubation with recombinant CagA (Fig. 1 panel f and Fig. 2 panel f).

As shown in Fig. 1 panel d, the expression for Bcl-2 was detected by immunoprecipitation in the MKN7 cells infected with *Hp cagA* and *vacA* positive strain, was significantly diminished during the time period of 3 h up to 48 h, with maximal inhibitory effect observed at 48 h of incubation. Densitometric analysis confirmed that the ratio of Bcl-2 protein to GAPDH protein was significantly reduced when MKN7 cells were incubated with or without recombinant CagA (Fig. 1 panel g and Fig. 2 panel g).

Figure 2 panel b shows that in the MKN7 cells which were incubated with a combination of *Hp* strain positive for *cagA* and *vacA* together with the addition of the recombinant CagA (10 μg per 1 ml of RPMI 1640 medium), the decrease in the signal intensity for HSP70 was not significantly different at 3 h and 24 h following the incubation as compared with that of *Hp* alone (Fig. 1 panel b) without the addition of CagA. However, when the incubation of the MKN7 cells with the *Hp* strain positive for *cagA* and *vacA* combined with exogenous CagA was prolonged up to 48 h, a complete disappearance of the signal for HSP70 expression was observed. The ratio of HSP70 protein over GAPDH protein confirmed that expression of HSP70 was significantly reduced at 3 and 24 h when compared with the control group and that the expression was not detectable at all, when this *Hp* strain was coincubated with exogenous CagA for 48 h (Fig. 2, panel e).

The expression of Bax was significantly increased at 24 and 48 h after the addition of CagA to the cells infected with Hp cagA vacA positive strain. The addition of exogenous CagA resulted in a significant decrease in Bcl-2 expression with the most pronounced effect observed at 48 h (Fig. 2, panels c and d).

### The effect of live Hp (cagA and vacA positive) and Hp (cagA and vacA negative) with or without coincubation with NS-398 on the COX-2 and Bax mRNA expression in MKN7 gastric epithelial cells

**Fig. 3:**
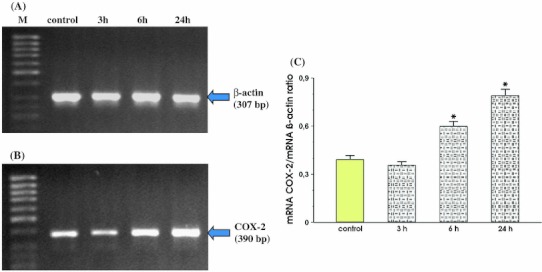
RT-PCR analysis of mRNA expression for COX-2 in the MKN7 cells exposed to *Hp* expressing *cagA* and *vacA*. **a** and **b** represent mRNA expression for β-actin and COX-2, respectively. Lane control means control cells (without infection). Following *lanes* represent mRNA expression for β-actin and COX-2 in MKN7 cells infected with *Hp* and incubated at 3, 6 and 24 h, respectively. **c** Represents the ratio of COX-2 mRNA over β-actin mRNA. Results are mean ± SEM of five experiments. *Asterisks* indicate a significant change (*p* < 0.05) as compared to control

In MKN7 cells infected with *Hp* positive for *cagA* and *vacA*, a significant increase in signal intensity for COX-2 mRNA at 3 h, 6 h and 24 h of incubation was recorded as compared to control cells (Fig. 3, panel b). The increase in COX-2 expression was first observed 3 h after *Hp* infection and the maximal rise of COX-2 mRNA expression was recorded at 24 h. The semi-quantitative ratio of COX-2 mRNA over β-actin mRNA confirmed that the expression of COX-2 reached the highest value in MKN7 cells incubated for 24 h with *Hp* strain positive for *cagA* and *vacA* (Fig. 3, panel c).

**Fig. 4:**
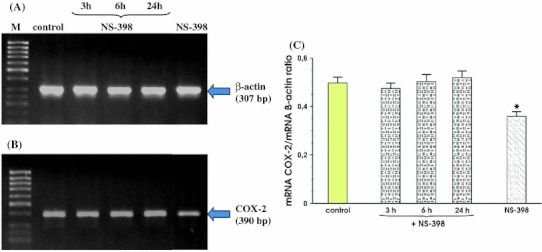
RT-PCR analysis of mRNA expression for COX-2 in the MKN7 cells exposed to *Hp* expressing *cagA* and *vacA* and coincubated with the selective COX-2 inhibitor, NS-398. **a** and **b** Represent mRNA expression for β-actin and COX-2, respectively. Lane control means control cells without *Hp* infection and without coincubation with NS-398. Following *lanes* represent mRNA expression for β-actin and COX-2 in MKN7 cells infected with *Hp* and incubated with NS-398 for 3, 6 and 24 h, respectively. Line NS-398 means MKN7 cells incubated with NS-398 only. **c** Represents the ratio of COX-2 mRNA over β-actin mRNA. Results are mean ± SEM of five experiments. *Asterisk* indicates a significant change (*p* < 0.05) as compared to control

Figure 4 shows that after 3 h, 6 h and 24 h of co-incubation MKN7 cells with *Hp* positive for *cagA* and *vacA* and NS-398, the signal for expression of mRNA for COX-2 was almost identical at each study period. Additionally, we found that incubation of MKN7 cells with NS-398 only, significantly reduced COX-2 mRNA in these cells at 24 h upon selective COX-2 inhibitor administration (Fig. 4, panel b). Densitometry analysis of mRNA expression presented as ratio of mRNA for COX-2 over mRNA β-actin confirmed that the signal intensity for COX-2 mRNA was similar in control cells and those incubated with *Hp* *cagA* and *vacA* positive with the addition of NS-398 at 3, 6 and 24 h following the beginning of incubation with this COX-2 inhibitor. This ratio was significantly decreased when MKN7 cells were incubated only with NS-398 (Fig. 4, panel c).

**Fig. 5:**
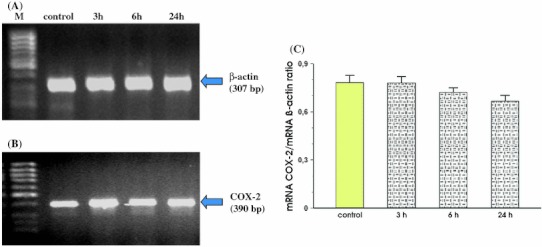
RT-PCR analysis of mRNA expression for COX-2 in the MKN7 cells exposed to *Hp cagA* and *vacA* negative. **a**, **b** Represent mRNA expression for β-actin and COX-2, respectively. Lane control means control cells without infection. Following *lanes* represent mRNA expression for β-actin and COX-2 in MKN7 cells infected with *Hp* and incubated at 3, 6 and 24 h, respectively. **c** Represents the ratio of COX-2 mRNA over β-actin mRNA. Results are mean ± SEM of five experiments

The effects of co-incubation of *Hp* strain positive for *cagA* and *vacA* with NS-398 were then compared with those for *Hp* negative for *cagA* and *vacA* applied alone or co-incubated with NS-398. For this purpose, the MKN7 cells were incubated with live *cagA* and *vacA* negative *Hp* with or without NS-398 for 3, 6 and 24 h, exactly as in the case of experiments with *Hp* strain positive for *cagA* and *vacA*. In contrast to the *Hp* *cagA* and *vacA* positive strain which caused a time-dependent rise in COX-2 expression, the signal for COX-2 mRNA did not change during co-infection of the MNK7 cells with *Hp* *cagA* and *vacA* negative strain and this was confirmed by the ratio of mRNA for COX-2 over mRNA β-actin (Fig. 5, panels b and c).

**Fig. 6:**
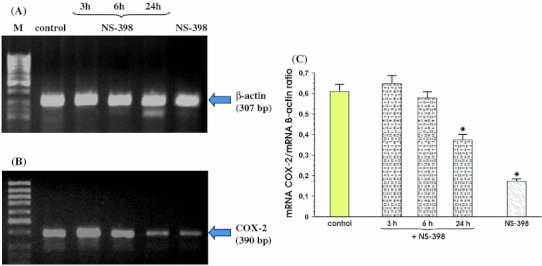
RT-PCR analysis of mRNA expression for COX-2 in the MKN7 cells exposed to *Hp cagA* and *vacA* negative strain and coincubated in the presence of NS-398. **a**, **b** represent mRNA expression for β-actin and COX-2, respectively. Lane Control means control cells without *Hp* infection and NS-398 addition. Following* lanes* represent mRNA expression for β-actin and COX-2 in MKN7 cells infected with *Hp* and co-incubated for 3, 6 and 24 h with NS-398. *Line* NS-398 means MKN7 cells incubated with NS-398 only. **c** Represents the ratio of COX-2 mRNA over β-actin. Results are mean ± SEM of 5 experiments. *Asterisks* indicate a significant change (*p* < 0.05) as compared to control

As illustrated in Fig. 6, panel b, COX-2 inhibitor, NS-398 failed affect COX-2 expression at 3 h and 6 h but significantly decreased mRNA expression for COX-2 at 24 h of incubation. Similarly as presented in Fig. 4, NS-398 significantly decreased the expression of COX-2 in MKN-7 cells non infected with *Hp*.

**Fig. 7:**
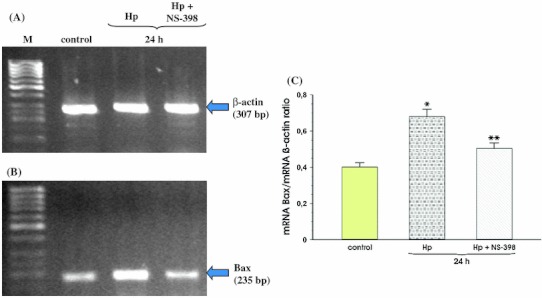
RT-PCR analysis of mRNA expression for Bax in the MKN7 cells exposed to *Hp* expressing *cagA* and *vacA* in the absence and the presence of NS-398. **a**, **b** Represent mRNA expression for β-actin and Bax, respectively. Lane control means control cells without *Hp* infection and NS-398 addition. Following* lane* represents mRNA expression for β-actin and Bax in MKN7 cells incubated 24 h with *Hp* and the next* lane* shows mRNA expression for β-actin and Bax in MKN7 cells incubated 24 h with the combination of *Hp* and NS-398. **c** Represents the ratio of Bax mRNA over β-actin mRNA. Results are mean ± SEM of five experiments. *Asterisks* indicate a significant change (*p* < 0.05) as compared to control. *Double asterisks* indicate a significant change (*p* < 0.05) as compared to MKN7 cell incubation with *Hp* alone

The expression of Bax mRNA increased 24 h after *Hp* infection and this effect was significantly inhibited by NS-398 (Fig. 7, panel b). Ratio of Bax mRNA over β-actin mRNA confirmed that the expression of Bax mRNA was significantly increased in cells infected with Hp strain positive for *cagA* and *vacA* and this effect was significantly ameliorated by co-incubation with selective COX-2 inhibitor (Fig. 7, panel c).

## Discussion

This study was designed to determine whether incubation of gastric mucosal cells with *Hp* influences the expression of HSP70, apoptosis related proteins Bax and Bcl-2 and expression of COX-2 in isolated gastric cells in vitro. Since the normal cultured human gastric epithelial cell line is not commercially available, we used for this purpose the gastric adenocarcinoma cell line MKN7, which is an established human gastric epithelium cell line for experimental studies in vitro conditions. Moreover, we attempted to determine the relationship between the apoptosis and COX-2 expression in this gastric adenocarcinoma cells infected with *Hp* strain and co-incubated with exogenous CagA to check whether recombinant CagA protein will aggravate the alterations in expression in HSP70, Bax and Bcl-2 proteins in the absence and presence of *Hp.*

In vitro studies using gastric cancer cell lines have shown that incubation of isolated gastric cells with either live or dead *Hp* or sonicates of *Hp* cells leads to an induction of apoptosis [31, 32] and results of our study with MKN7 cells are in agreement with these observations. At present, the mechanism by which *Hp* induces apoptosis in gastric cancer cells and interacts with HSP70 and COX-2 expression remains unknown.

There is increasing evidence that apoptosis plays an important role in the pathogenesis of a variety of infectious diseases. Although apoptosis as a process of cell death occurs in normal mucosal cells under physiological conditions, the excessive apoptosis results in tissue damage. We extended our and others previous observation in vivo [33, 34] that *cagA* positive *Hp* strains induce apoptosis as evidenced by increasing expression of pro-apoptotic Bax and decreasing expression for anti-apoptotic Bcl-2. In addition, CagA negative *Hp* strain also enhanced apoptosis suggesting that CagA is not a prerequisite for apoptosis to occur as suggested previously [35]. This notion is to some extent in keeping with observation by Peek et al. [36] and Zhang et al. [37] who reported that in patients infected with *cagA* positive *Hp* strains the apoptotic index was lower than in those with *cagA* negative *Hp* strains. In clear contrast, the gastric infection with *Hp* *cagA* positive strains was shown to induce an overexpression of proapoptotic proteins in the gastric mucosa [38, 39], similarly as in our present study with transformed cell line.

Most in vitro and animal studies have shown that both *Hp* cagA positive and *Hp* cagA negative strains enhanced apoptosis sometimes with no relation to CagA expression [27, 34, 37, 38] suggesting that our results with *Hp* infection of cancer cell line seems to mimic those obtained in normal gastric epithelial cells with respect to apoptosis at early time after infection. For example, Ashktorab et al. [38] reported that infection of gastric cells with a cagA positive *Hp* strain resulted in Bax translocation to mitochondria, caspase-3 activation, and ultimately cell death. Interestingly, *Hp* CagA positive strain induced apoptosis at 2 weeks after inoculation but this increased apoptosis almost declined to the baseline level after 12 months of inoculation in rats [40]. Therefore, it is not excluded that the early enhancement in apoptosis process in both, normal and cancer cells may involve the formation of monochloramine (NH_2_Cl) observed in normal rat gastric epithelial cell line RGM-1 [41] and in gastric cancer cells MKN45 [42].

In addition besides CagA another cytotoxin of *Hp*, VacA, has been reported to induce apoptosis [43]. Purified, recombinant VacA caused fragmentation of DNA in human gastric adenocarcinoma AGS cell line [13]. MKN7 cells were incubated with *Hp* expressing *cagA* and *vacA* and supplemented with exogenous CagA, the trend in protein expression of Bax and Bcl-2 was similar to that observed in cells infected with *Hp* without CagA supplementation suggesting that exogenous CagA itself exerts not a direct stimulatory influence on gastric epithelial cells apoptosis. However, the most pronounced effect on apoptosis was observed when exogenous CagA was added to *Hp* positive for *cagA* and *vacA* cytotoxins suggesting that action of CagA may involve indirect effect, e.g. inhibition of HSP70. Indeed, CagA injected by *Hp* into the host epithelial cells was shown to “hijack” physiological signal transduction and caused pathological cellular response such as increased cell apoptosis, proliferation and morphology changes [26]. Our study indicates that this promotion of cell apoptosis by *Hp* strain positive for *cagA* and *vacA* is also evident in epithelial adenocarcinoma cell line MKN7. In addition, the CagA by itself can induce ROS production causing DNA damage, thus contributing to enhanced apoptosis in early stages of gastric carcinogenesis.

**Fig. 8:**
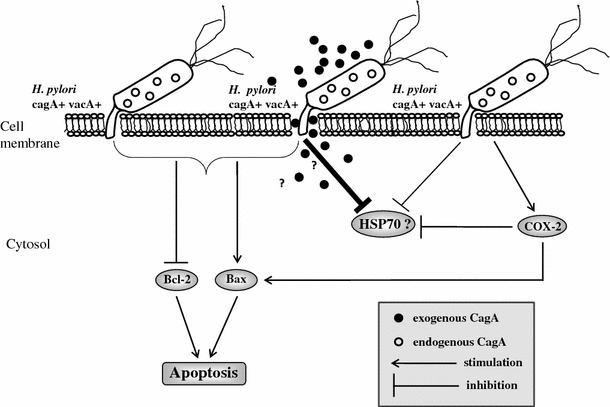
Schematic hypothesis presenting model of *Hp positive cagA and vacA strain* interaction with human gastric epithelial MKN7 cells *via* enhancement of apoptosis, the inhibition of HSP70 and activation of COX-2 by this bacteria and its cytotoxin CagA

The major finding of our present study is that *Hp* can directly attenuate the expression of HSP70 in MKN7 cells which could by associated with the increase in apoptosis. Specific downregulation of HSP70 in MKN7 cells was observed, especially under condition where the live *Hp* expressing *cagA* and *vacA* was added to the cell culture and incubated for the period of 48 h. Moreover, when the exposure of the cells with *Hp* strain positive for *cagA* and *vacA* was combined with exogenous CagA administration, a complete disappearance of the signal of HSP70 mRNA was recorded, suggesting that the addition of CagA to the cells infected with *Hp* positive for cagA and vacA, accelerated the inhibitory effect of bacteria and its cytotoxin on HSP70 expression (see, our hypothesis presented in Fig. 8). It should be noted, however, that cell incubation with recombinant CagA alone failed to affect the expression of mRNA for HSP70 (not shown), suggesting that the CagA protein itself does not exhibit inhibitory influence on HSP70 expression but exerts this inhibition only in the presence of *Hp*. This is corroborative with the observation [43, 44] that the genes of the cag *PAI* actually encode for a functional type IV secretion system, by which *Hp* delivers the CagA protein directly into gastric epithelial cells. The possible molecular mechanism of the attenuation of HSP70 expression by *Hp* involves TFSS (type IV secretion system) and the subsequent activation of STAT3 (signal transducers and activators of transcription 3) pathway and activation of caspase-3 dependent apoptosis pathway as has been recently proposed by our group, with regards to another human gastric epithelial cell line [45].

We conclude that *Hp* action involves the “injection” of CagA into the eukaryotic cells, or another not yet recognized mechanism related to the damage of cellular membrane and/or inhibitory effect of bacteria on tight by recruiting ZO-1 [46] that impaired cell junctions allowing for the transport of cytotoxin such as CagA by this bacteria and/or in the presence of this germ. Our working hypothesis is that *Hp* possesses an ability to deliver biologically active proteins such as CagA into eukaryotic cells, because the most pronounced inhibitory effect of *Hp* on HSP70 was observed in MKN7 cells additionally co-incubated with CagA. Our findings are, however, limited to the gastric adenocarcinoma cell line and further studies are needed to confirm this hypothesis in normal human gastric epithelial cells.

Our finding that *Hp* expressing cagA and vacA exerts direct effect on the structure of the epithelial cells causing its increased fragility and inhibits HSP70 expression suggests that downregulation of HSP70 and lack of its protective influence on cell defense system, might be one of the cause of many other *Hp*-associated alteration of the Bax/Bcl2 cellular equilibrium (Fig. 8). In normal rat gastric epithelial cells (RGM-1) *Hp* significantly attenuated the expression HSP70, whereas exposure of the *Hp*-infected cells to short non-cytotoxic heat shock or geranylgeranylacetone (GGA) restored HSP70 expression in RGM-1 cells [47, 48]. *Hp* upregulates COX-2 mRNA expression in gastric cancer cells, and both premalignant and malignant gastric lesions demonstrated high COX-2 expression levels in vivo, suggesting that, *Hp*-induced gastric carcinogenesis is associated with elevated expression of COX-2 in neoplastic gastric epithelium [49–51]. Herein, we have also demonstrated, that both *Hp* strains, namely *Hp* *cagA* and *vacA* positive and *Hp* *cagA* and *vacA* negative caused an up-regulation of COX-2 mRNA in MKN7 cells and this effect was inhibited by NS-398. Likewise, the NS-398 alone markedly inhibited the mRNA expression for COX-2 whether or not cells were infected with *Hp cagA* and *vacA* negative strain. Since COX-2 overexpression is involved in gastric carcinogenesis, the suppression of COX-2 expression and activity might be useful in a chemoprevention strategy [52]. In our present in vitro study, NS-398 not only inhibited the upregulation of mRNA expression for COX-2 in *Hp*-infected MKN7 cells, but also suppressed Bax expression. Li et al. [53] also showed that the selective COX-2 inhibitor attenuated the cell proliferation and apoptosis of the human gastric cancer cell line BGC-823, which may be attributed to the inhibition of cell cycle progress. On the other hand, Konturek et al. [54] reported that in gastric cancer patients 14-day treatment with selective COX-2 inhibitor a significant up-regulation of caspase-3 mediating apoptotic cell death was observed. This indicates that *Hp* apparently promotes an apoptosis in vitro at early stage of infection but could exert opposite effect in vivo in long term infected patients with chronic and atrophic gastritis. COX-2 derived products and COX-2 inhibitors may differ in apoptotic response as to whether apoptosis is determined in isolated cells or in vivo condition.

In summary, we found that co-incubation of MKN7 epithelial cells with *Hp* without or with addition of CagA changed the equilibrium between Bax and Bcl-2 expression towards proapoptotic state (Bax) while simultaneously decreasing expression for anti-apoptotic Bcl-2. Thus it is hypothesized (Fig. 8) that besides HSP, also the PG generated *via* COX-2 expression and activity could be considered as another important activator of apoptosis triggered by *Hp* in the presence of CagA cytotoxin.
